# ﻿A new species of the genus *Yoldiella* (Bivalvia, Protobranchia, Yoldiidae) from Haima Cold Seep, South China Sea, China

**DOI:** 10.3897/zookeys.1204.121088

**Published:** 2024-06-06

**Authors:** Qi Gao, Yan Tang, Junlong Zhang

**Affiliations:** 1 School of Life Sciences, Qingdao Agricultural University, Qingdao 266109, China Institute of Oceanology, Chinese Academy of Sciences Qingdao China; 2 Laboratory of Marine Organism Taxonomy and Phylogeny, Qingdao Key Laboratory of Marine Biodiversity and Conservation, Institute of Oceanology, Chinese Academy of Sciences, Qingdao 266071, China Qingdao Agricultural University Qingdao China; 3 Marine Biological Museum, Chinese Academy of Sciences, Qingdao 266071, China Marine Biological Museum, Chinese Academy of Sciences Qingdao China; 4 University of Chinese Academy of Sciences, Beijing 100049, China University of Chinese Academy of Sciences Beijing China

**Keywords:** Anatomy, chemosynthetic ecosystems, deep-sea, molecular analysis, morphology

## Abstract

In present study, a previously unidentified but frequently encountered species of deep-sea protobranch, *Yoldiellahaimaensis***sp. nov.**, is described new to science from the Haima Cold Seep on the northwestern slope of the South China Sea. A morphological analysis confirmed that this species belongs to a previously undescribed species of the genus *Yoldiella* A.E. Verrill & K.J. Bush, 1897. It differs morphologically from other known species within the genus in its shell shape, degree of inflation, beaks, and number of hinge teeth. Furthermore, we sequenced three gene segments of *Y.haimaensis***sp. nov.**, comprising a nuclear ribosomal gene (18S rRNA), a nuclear protein-coding gene (histone H3), and a mitochondrial gene (cytochrome c oxidase subunit I, COI). Our phylogenetic analysis performed on the superfamily Nuculanoidea and family Yoldiidae indicates that the genus *Yoldiella* is non-monophyletic, and the widely recognized families within the superfamily Nuculanoidea are also not monophyletic. Our results provide molecular insights into the Protobranchia and highlight the necessity for further samples and data to revise the classification of families and genera within the superfamily using an integrative approach that combines morphological analysis and molecular data.

## ﻿Introduction

Protobranchia, with a significant evolutionary history dating back to the Cambrian, represents an ancestral and basal group of Bivalvia. The protobranchs are primarily found in the subtidal zone, especially in the deep sea, and are generally deposit feeders that bury themselves in the soft sediment (Allen 1978). They have limited presence in the intertidal zone. So, it is difficult to collect specimens of this group (Xu 1999). The highly conserved and distinctive morphology and anatomy, including gill structure, hinge conformation, shell microstructure, as well as the pericalymma larval development, small in size and lifestyle of the group make it a difficult but fascinating taxon of Bivalvia (Zardus 2002). The simplicity in the form of protobranch bivalves veils the complexity of their phylogeny. The monophyly of Protobranchia has been discussed intensively and become a subject of controversy due to the extensive use of molecular methods (Smith et al. 2011; Sharma et al. 2012; Bieler et al. 2014; González et al. 2015; Combosch et al. 2017; Lemer et al. 2019). Phylogenetic analyses using four nuclear genes (Sharma et al. 2012) and an exemplar-based approach combining Sanger-based sequences and an extensive morphological data matrix (Bieler et al. 2014), as well as the phylogenomic analysis using genomes and transcriptomes (González et al. 2015), recovered the monophyly of Protobranchia. However, the subsequent analysis by Combosch et al. (2017), which utilized five genes and included more taxa, supported the polyphyly of Protobranchia. The latest research has divided Protobranchia into three orders and five superfamilies: Nuculanoidea and Sareptoidea in Nuculanida, Solemyoidea and Manzanelloidea in Solemyida, and Nuculoidea in the order Nuculida (Sharma et al. 2013; Sato et al. 2020). The reconstruction of the phylogeny has indicated that eight families (i.e. Nuculanidae, Bathyspinulidae, Malletiidae, Neilonellidae, Phaseolidae, Siliculidae, Tindariidae, and Yoldiidae) within the superfamily Nuculanoidea are all non-monophyletic (Sato et al. 2020). Shell microstructure of protobranchs play a crucial role in their classification at the superfamily level. Moreover, subtle differences in shell microstructure can aid in distinguishing similar species (Sato et al. 2020). Extensive research has revealed a multitude of unknown or cryptic protobranch species awaiting description and classification under the integrative taxonomy framework (e.g. Neulinger et al. 2006; Zardus et al. 2006). Additional samples and data are required to revise the families and genera within the superfamily Nuculanoidea through a combination of morphological diagnosis and molecular analysis.

Cold seeps are natural phenomena widely distributed across the globe. On the seafloor of these areas, the hydrocarbon-rich fluids and gases leak from cracks and enter the water column through sediment, forming a distinctive habitat (Dong et al. 2021). In March 2015, a newly active cold seep was discovered using the *Haima* remotely operated vehicle (ROV) on the northwestern slope of the South China Sea (SCS) (Zhao et al. 2020). Dong et al. (2021) documented 34 epibenthic macrofauna species collected from Haima Cold Seep with 24 species being identified. Yao et al. (2022) identified 12 macrobenthic species from five phyla and 12 families in the Haima Cold Seep, including two species first found in this location. Seven phyla, 14 classes, and 65 species were identified by He et al. (2023) in this cold seep. To date, more than 80 species of macrobenthic organisms have been collected from the Haima cold seeps (Wang et al. 2022).

This study presents the description of a new *Yoldiella* species, *Y.haimaensis* sp. nov., from the Haima Cold Seep in the SCS. This species had been identified as *Malletia* sp. or *Yoldiella* sp. (Dong et al. 2021; Ke et al. 2022; He et al. 2023). Additionally, we provide the sequences of three gene segments of the new species, including a nuclear ribosomal gene (18S rRNA), a nuclear protein-coding gene (histone H3), and a mitochondrial gene (cytochrome c oxidase subunit 1, COI). A phylogenetic analysis was conducted on the superfamily Nuculanoidea and family Yoldiidae, providing molecular data for the study of Protobranchia and enhancing understanding of macrobenthos at the Haima Cold Seep.

## ﻿Materials and methods

### ﻿Specimen collection and identification

**Figure 1.**
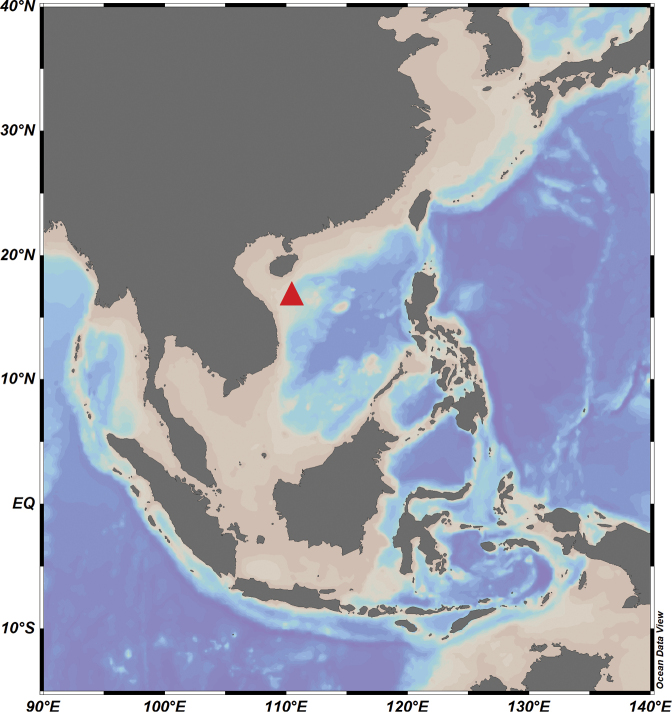
Map of samples site (triangle) of *Yoldiellahaimaensis* sp. nov.

The specimens were collected on July 4–12, 2022 from the Haima Cold Seep at a water depth of 1390 m in the SCS (16°43'N, 110°28'E) (Fig. 1) using the ROV and TV Grab of Research Vessel *Kexue*. All specimens were fixed in 100% ethanol and deposited at the Marine Biological Museum (**MBM**), Chinese Academy of Sciences, Qingdao, China.

### ﻿DNA extraction, amplification, and sequencing

Two specimens with tissues were randomly selected from the specimens collected. DNA was extracted from the muscle tissues using the TIANamp Marine Animals DNA Kit. Three gene fragments were amplified, including a nuclear ribosomal gene (18S rRNA), a nuclear protein-coding gene (histone H3), and a mitochondrial gene (cytochrome c oxidase subunit 1, COI), which were subsequently utilized for phylogenetic analyses. The PCR program for the mitochondrial genes was as follows: initial denaturation for 180 s at 94 °C, followed by 35 cycles of denaturation for 30 s at 94 °C, annealing for 45 s at 46 °C and elongation for 60 s at 72 °C. The final elongation step was conducted for 10 min at 72 °C. The PCR conditions for the 18S rRNA and H3 genes were performed the same as above, with the only difference being in the annealing step. The annealing temperature for H3 was 55 °C, and the annealing step for 18S rRNA was performed in a touch-down manner (Don et al. 1991) with an initial annealing temperature of 70 °C followed by a reduction of 1 °C per cycle until 65 °C. The primer sequences are listed in Suppl. material 1.

The PCR products were separated by electrophoresis using 1.0% agarose gels, purified, and then sent for sequencing to Sangon Biotech Co. Ltd.

### ﻿Phylogenetic analyses

The sequences newly acquired in this study and obtained from NCBI are listed in Suppl. materials 2, 3. All sequences were aligned using MAFFT v. 7 software (Katoh and Standley 2013) based on the amino acid sequences and employing the Auto and G-INS-I algorithms. Subsequently, the aligned sequences in each dataset were manually trimmed to the same length. Ambiguously aligned sites in the ribosomal gene were removed using GBLOCKS v. 0.91b (Castresana 2000) with the least stringent settings. Because of the limited molecular data of Protobranchia, different combined gene datasets were used for phylogenetic analyses of the family Yoldiidae and superfamily Nuculanoidea. The final dataset for Yoldiidae phylogenetic trees comprised 1059 bp of 18S rRNA (88% of 1192 bp before Gblock), and 594 bp of COI. The final dataset for Nuculanoidea phylogenetic trees included 1101 bp of 18S rRNA (81% of 1352 bp before Gblock), 588 bp of COI, and 305 bp of H3. The COI, 18S rRNA, and H3 gene sequences from the same individuals were concatenated using SequenceMatrix software (Vaidya et al. 2011) to form a combined gene dataset. The Maximum likelihood (ML) and Bayesian inference (BI) analysis based on concatenated datasets of COI and 18S rRNA were used for phylogenetic analyses. The ML tree was conducted using IQ-TREE v. 2.2.0-Linux (Nguyen et al. 2015). The most suitable evolution model was found by ModelFinder (Kalyaanamoorthy et al. 2017) and adopted automatically to infer the ML tree. Bootstrap supports (BS) were calculated with 1,000 replicates to assess branch supports. Gene partition models chosen for IQ-TREE were 18S rRNA, K2P+I; COI, TIM+F+I+G4; and combined gene, TIM3+F+I+G4. jModelTest v. 2.1.10 (Darriba et al. 2012) was used to evaluate the best-fitting nucleotide substitution model and derive the optimal model of the Bayesian phylogenetic tree. According to the Akaike information criterion (AIC), the best model for each isolated gene was 18S rRNA, HKY+I; COI, GTR+I+G; and combined gene, HKY+I+G. A Bayesian-inference (BI) analysis was performed using MrBayes v. 3.2.6 (Sharma et al. 2013) and the best model of each dataset. The posterior probability (PP) was estimated using four chains running 1 million generations and sampled every 100 generations. The first 25% of sampled trees were discarded as burn-in. The results of ML and BI trees were visualized and rendered using Figtree v. 1.4.4 (http://tree.bio.ed.ac.uk/software/figtree/).

### ﻿Species delimitation

A variety of species delimitation methods were employed to determine that the species described here is not conspecific with another, already known species of Yoldiidae. COI data were analyzed using the program Automated Barcode Gap Discovery (ABGD; Puillandre et al. 2012) and Assemble Species by Automatic Partitioning (ASAP; Puillandre et al. 2021), a method to build species partitions from single-locus sequence alignments. Single gene trees were analyzed by applying the Bayesian implementation of the Poisson Tree Processes model (bPTP; Zhang et al. 2013) at the web server of the Heidelberg Institute for Theoretical Studies, Germany (http://species.h-its.org/).

## ﻿Results

### ﻿Systematics

**Subclass Protobranchia Pelseneer, 1889**

**Order Nuculanida J.G. Carter, D.C. Campbell & M.R. Campbell, 2000**

**Superfamily Nuculanoidea H. Adams & A. Adams, 1858 (1854)**

**Family Yoldiidae Dall, 1908**

#### ﻿Genus Yoldiella

Taxon classificationAnimaliaNuculanidaYoldiidae

A.E. Verrill & K.J. Bush, 1897

EF1A454B-FA93-50DB-AF61-C0AB23DECF1F

##### Type species.

*Yoldiellalucida* (Lovén, 1846) (International Commission on Zoological Nomenclature 1985: Opinion 1306) by original designation (Recent, North Atlantic).

#### ﻿ Yoldiella haimaensis sp. nov.

Taxon classificationAnimaliaNuculanidaYoldiidae

9402E99D-F05E-5108-B6D8-DE9D880F5C62

https://zoobank.org/CCA23CFC-0081-4096-BD4C-98ACEB76DD8F

Figs 2 , 3 , 4

Malletia sp.: Dong et al. 2021: 4, fig. 5b; Ke et al. 2022: 4, fig. 2h.
Yoldiella sp.: He et al. 2023: 6, fig. 2Q.

##### Type specimens.

***Holotype***: MBM 229041: length 7.5 mm, width 3.2, height 5.1 mm. Paratypes: MBM 229042: length 7.4 mm, width 3.3 mm, height 5.0 mm; MBM 229043: length 6.5 mm, width 2.6 mm, height 4.4 mm; MBM229044: length 6.3 mm, width 2.5 mm, height 4.1 mm; MBM229045: length 7.6 mm, width 3.0 mm, height 4.9 mm.

##### Type locality.

Haima Cold Seep (depth 1390 m, 16°43.00'N, 110°28.00'E), off southern Hainan Island, South China Sea.

##### Diagnosis.

*Yoldiellahaimaensis* sp. nov. differs morphologically from other known species within the genus in shell shape, degree of inflation, beaks, and number of hinge teeth. Diagnostic characteristics: shell small, ovate, inflated medially. Posterior end slight produced. Resilifer triangular, projecting. Beak rather lower than other species, suborthogyrate, and easily worn. Hinge plate narrow; posterior hinge plate smaller than anterior one, with taxodont teeth in two series; 17–19 anterior and 15–16 posterior teeth on hinge plate.

**Figure 2.**
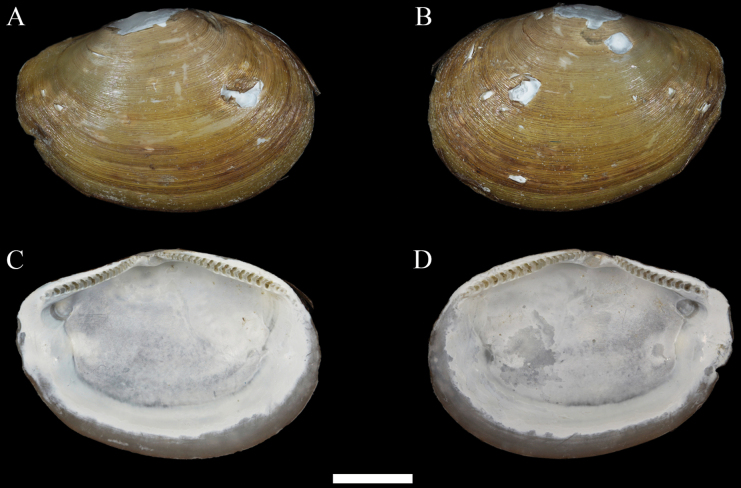
*Yoldiellahaimaensis* sp. nov. **A–D** holotype, MBM229041. Scale bar: 2 mm (all at the same scale).

##### Description.

Shell small, elongate, ovate in outline, moderately inflated, opaque, fragile, 2.2–8.2 mm long, W/L about 0.40; H/L about 0.66, usually subequivalve, inequilateral. Shell surface smooth, with numerous very fine, regular, and nearly isometric growth lines, without radial stria. Periostracum light brown and flaky. Umbo slightly posterior to middle, low, large, obscure, opisthogyrate, and easily worn. Antero-dorsal margin convex; anterior end broadly rounded, merging smoothly to ventral margin. Ventral margin slightly convex, with very shallow sinus at postero-ventral corner. Postero-dorsal margin oblique and then convex, descending to blunt posterior margin. Posterior end slight produced. Escutcheon and lunule obscure. ligament amphidetic, thin, short.

**Figure 3.**
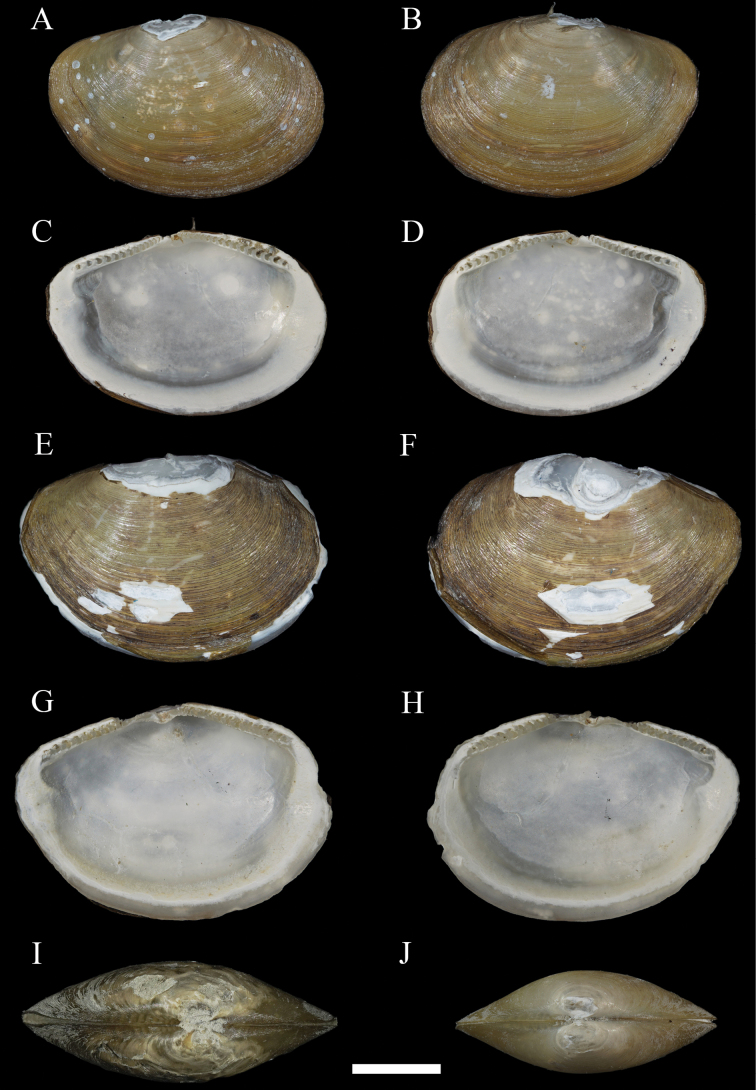
*Yoldiellahaimaensis* sp. nov. **A–D** paratype 1, MBM229042 **E–H** paratype 2, MBM229043 **I** paratype 3, MBM229044 **J** paratype 4 MBM229045. Scale bar: 2 mm (all at the same scale).

Internal surface porcelaneous white. Hinge plate moderately broad, narrow below umbo, moderately long, and rather strong, with two chevron-shaped columns and moderately sized taxodont lateral teeth, about 17–19 anterior teeth, about 15–16 posterior teeth, interrupted by a triangular, projecting resilifer, and not extend beyond the inner limit of adductor muscles. Angle of about 140° between anterior and posterior hinge plates. Posterior hinge plate usually smaller than anterior. Resilium oblique and often obscure in dry preserved specimens. Adductor scar obscure to evident; triangular anterior adductor scar larger than droplet-shaped posterior adductor scar. Pallial sinus obsolete; pallial line usually entire.

**Figure 4.**
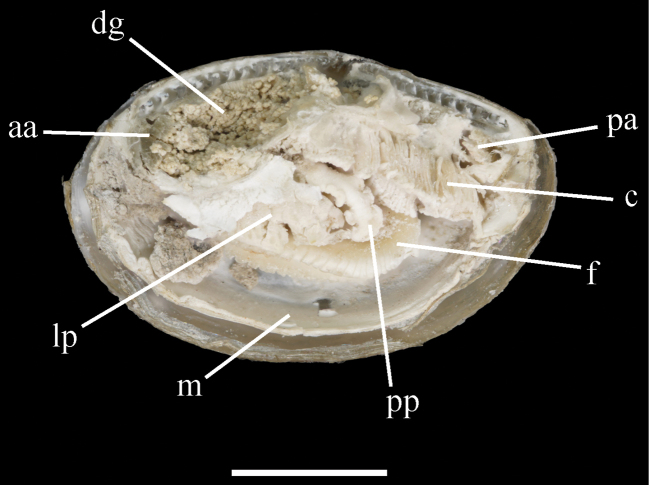
Anatomy features of *Yoldiellahaimaensis* sp. nov. **aa**, anterior adductor; **c**, ctenidium; **f**, foot; **lp**, labial palp; **m**, mantle; **pa**, posterior adductor; **pp**, Palp proboscis; **dg**, digestive gland. Scale bar: 2 mm.

Mantle large, thin, and opaque; anterior adductor crescent-shaped, twice or three times size of posterior. Ctenidium structure simple and lamellar, at posterior side parallel to the postero-dorsal shell margin. Labial palp size moderate, consisting of flat, paired lamellae on each side, with appendages of elongated palp proboscis. Foot muscular and large, with a regular series of nearly rectangular protrusions at margins, partially covered by labial palp. Siphons combined posteriorly.

##### Etymology.

The species epithet “*haimaensis*” is Latin and means “from Haima”, which refers to the name of the cold seep where the specimens were collected.

##### Distribution.

Currently known only from the Haima Cold Seep on the northwestern slope of the South China Sea.

##### Remarks.

*Yoldiellahaimaensis* sp. nov. differs morphologically from other known species of *Yoldiella* in its shell shape, degree of inflation, beak characteristics, and number of hinge teeth. Its beaks are lower than those of other species, suborthogyrate, and prone to wearing easily. This new species resembles the type species of *Yoldiella*, *Y.lucida*. However, *Y.haimaensis* sp. nov. differs from *Y.lucida* in having lower, suborthogyrate beaks that wear easily and a slightly rounded posterior end. *Yoldiellahaimaensis* sp. nov. has more teeth (17–19 anterior teeth, 15–16 posterior teeth) than *Y.lucida* (8 teeth on each end), with more anterior teeth than posterior teeth. Another species closely resembling *Y.haimaensis* sp. nov. in outline is *Yoldiellasagamiana* T. Okutani & K. Fujikura, 2022 from Sagami Bay, but *Y.sagamiana* has a larger W/L ratio and fewer teeth (15 anterior teeth, 10 posterior teeth) than the new species, and *Y.sagamiana* also has more pointed beaks and finer commarginal cords and lines. The outline of the new species is similar to *Yoldiellabiguttata* Allen, H. L. Sanders & F. Hannah, 1995 from the Guyana Basin, but *Y.biguttata* has the more prominent umbo and the anterior and posterior series are either equal or with the anterior series having one additional tooth (5–6 in the largest specimen).

### ﻿Species delimitation

**Figure 5.**
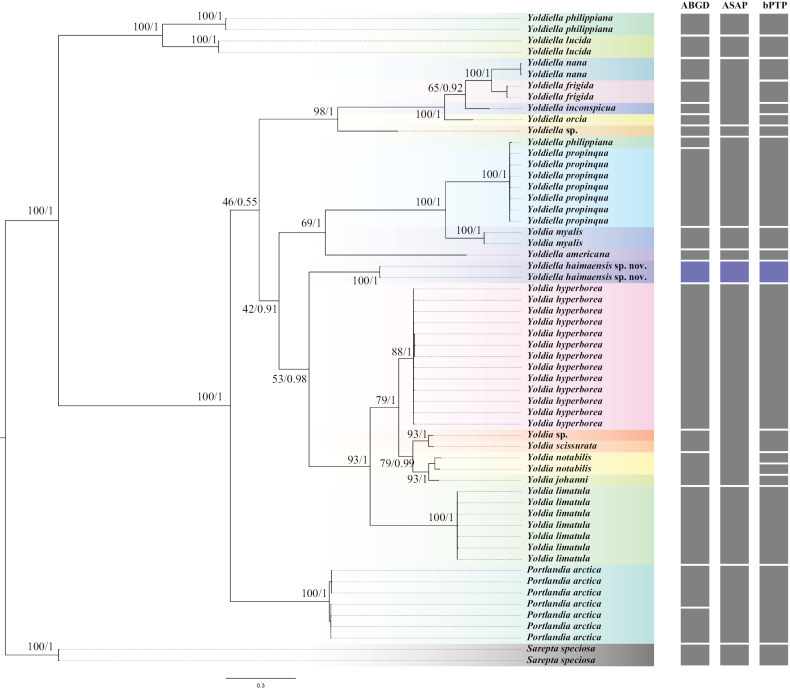
Phylogenetic tree inferred by Bayesian inference (BI) and maximum likelihood (ML), and species delimitation based on COI gene. Numbers adjacent to nodes refer to ML bootstrap scores and BI posterior probability (left, and right, respectively). The results of three species delimitation methods are shown on the right of phylogenetic tree (each rectangle represents one species).

Our species delimitation using the ABGD, ASAP, and bPTP methods show slightly different results, but they all support that our two samples are the same new species, and distinct from others in the family Yoldiidae (Fig. 5). ABGD and bPTP delineated the data into 19 species, while ASAP delineated the data into 12 species. ASAP grouped *Yoldiellanana* (M. Sars, 1865), *Yoldiellafrigida* (Torrell, 1859), *Yoldiellainconspicua* A.E. Verrill & K.J. Bush, 1898, and *Yoldiellaorcia* (Dall, 1916) into one species; ASAP and bPTP grouped *Yoldiellaphilippiana* (Nyst, 1845) and *Yoldiellapropinqua* (Leche, 1878), which clustered together, into one species; ABGD and bPTP grouped *Yoldia* sp. and *Yoldiascissurata* Dall, 1897 into one species; ABGD grouped *Yoldianotabilis* Yokoyama, 1922 and *Yoldiajohanni* Dall, 1925 into one species; bPTP delineated two species for the two individuals of *Y.notabilis*; ASAP grouped *Yoldiahyperborea* (A. Gould, 1841), *Yoldia* sp., *Y.scissurata*, *Y.notabilis*, and *Y.johanni* into one species; ABGD delineated two species for the seven individuals of *Portlandiaarctica* (Gray, 1824).

### ﻿Molecular phylogenies

**Figure 6.**
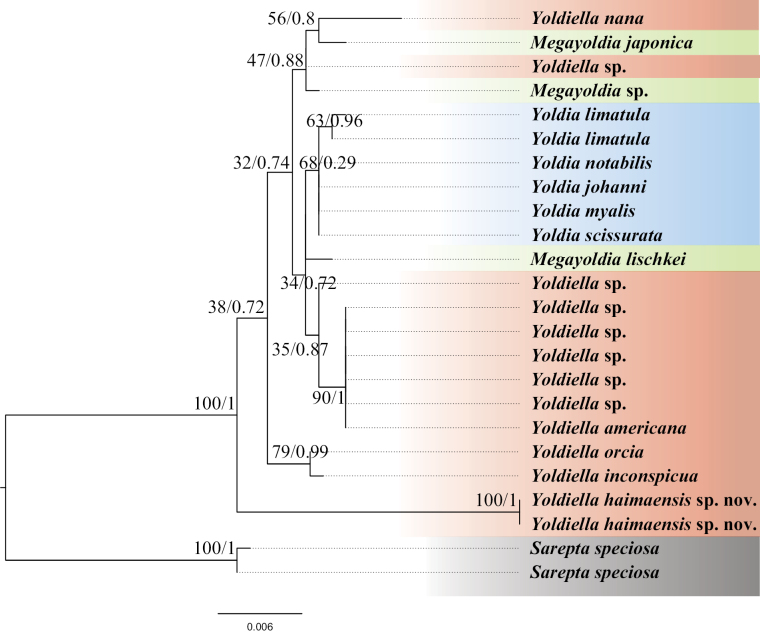
Phylogenetic tree inferred by Bayesian inference (BI) and maximum likelihood (ML) based on 18S rRNA gene. Numbers adjacent to nodes refer to ML bootstrap scores and BI posterior probability (left, and right, respectively).

Due to the limitation of molecular data of Protobranchia, only COI and 18S rRNA could meet the requirements of the phylogenetic tree construction of the family Yoldiidae, and their data were different, so these genes were used to construct two separate phylogenetic trees (Figs 5, 6). *Sareptaspeciosa* A. Adams, 1860 from the closely related superfamily Sareptoidea was selected as the outgroup in our phylogenetic analyses. BI and ML analyses yielded similar results. Phylogenetic analyses based on COI and 18S rRNA both showed that *Yoldiella* is not monophyletic. However, there were some differences. In the COI phylogenetic analysis, *Y.nana* and *Y.frigida* clustered into a sister taxon and has a close relationship with *Y.inconspicua* and *Y.orcia*, successively, while in 18S rRNA phylogenetic analysis, *Y.nana* clustered with species of the genus *Megayoldia* A.E. Verrill & K.J. Bush, 1897, and was distantly related to *Y.inconspicua* and *Y.orcia*. In addition, the COI analysis revealed that species of the genus *Yoldia* Møller, 1842 were nested among species of *Yoldiella*. Conversely, in the phylogenetic analysis constructed using 18S rRNA, *Yoldia* clustered as a distinct branch. The phylogenetic status of *Y.haimaensis* sp. nov. was different owing to the incongruent results of separate molecular analyses. The phylogenetic analysis based on COI showed that the new species was positioned at the base of the clade including species of *Yoldia* (Fig. 5), while it was at the basal position of the clade containing all *Yoldiella*, *Yoldia*, and *Megayoldia* species in the 18S rRNA phylogenetic tree with low support values (Fig. 6).

Phylogenetic analysis constructed using a combined gene dataset (COI+18S+H3) did not improve resolution as more taxa were added within the superfamily Nuculanoidea (Suppl. materials 4, 5). The BI and ML analyses recovered different topologies, and most nodes at high levels received very low support values. Furthermore, the results indicate that the family Yoldiidae is non-monophyletic, and that none of the widely recognized families (Nuculanidae, Neilonellidae, Malletiidae, Siliculidae, Phaseolidae, Tindariidae, and Bathyspinulidae) form monophyletic groups in this reconstruction.

## ﻿Discussion

The genus *Yoldiella* is believed to be ubiquitous in all the world’s oceans, with a particularly high abundance in deep waters (Benaim and Absalão 2011a). Environmental differences are likely to have a significant impact on the weight of their shell and the rate of their growth (Reed et al. 2013). However, their small size and offshore habitat contribute to their rarity in collections. Prior to this study, no species of this genus had been identified in the South China Sea. The discovery of *Y.haimaensis* sp. nov. bridges this geographical gap. In fact, this new species has been encountered more than once during the investigation and research on cold-seep fauna in the South China Sea, but in these studies, it has been misidentified as *Malletia* sp. or as *Yoldiella* not to species (Dong et al. 2021; Ke et al. 2022; He et al. 2023). The detailed description of *Y.haimaensis* sp. nov. contributes to the understanding of macrobenthos in the Haima Cold Seep.

*Yoldiella* is a difficult taxon to define because the morphological differences within this genus are mostly subtle and there are many closely related species. This genus was established by Verrill and Bush (1897), and it is usually characterized by ovate or wedge-shaped shells, which always have a slight postero-ventral sinuosity. The internal cartilage is often relatively large and occupies a simple notch. The external ligament is weak, and the pallial sinus is usually indistinct. Since then, its classification and composition have been controversial. Allen and Hannah (1986) and Allen et al. (1995) redefined the genus *Yoldiella* considering shell shape, hinge morphology, musculature, and the extent and course taken by the hindgut, which have been widely accepted by researchers. The redescription of *Yoldiella* by Coan and Valentich Scott (2012) limited it to forms having an elongate, amphidetic ligament with an internal section, an obscure or absent escutcheon and lunule, and a small pallial sinus.

It is widely acknowledged that soft-tissue analysis plays a crucial role in contemporary malacology. However, there is a significant lack of detailed morphological descriptions for this taxon. Yonge and Calman (1939) presented a comprehensive summary of the anatomical characteristics of Protobranchia. Purchon (1956) surveyed the structure and function of the stomach throughout the Protobranchia to establish evidence for phylogenetic relationships between the families and orders within the subclass. Xu (1999) highlighted a significant distinction in the anatomical features of Protobranchia compared to other bivalves, emphasizing the muscular nature of their feet for crawling. The ctenidium assumes a lamellar shape primarily for respiratory purposes, while the well-developed labial palp functions as a distinct feeding organ. Okutani and Fujiwara (2005) illustrated the soft part of *Yoldiellakaikonis* Okutani & Fujiwara, 2005. Reed et al. (2014) described and deliberated on the gonad morphology and oocyte size of four species of *Yoldiella*. Yonge and Calman (1939) and Allen et al. (1995) provided detailed accounts of the anatomical characteristics of *Y.lucida*, the type species of *Yoldiella*, noting that the organs in this species are more compactly arranged due to its relatively shorter shell, which exhibits a greatly abbreviated posterior rostrum. The labial palps are moderately large and extended, with well-developed, long, and muscular palp proboscides. The foot is relatively larger, as are the ctenidia, the filaments of which are broader and deeper. The shell and anatomical features of the new species align with the aforementioned characteristics of *Y.lucida*.

The genus *Yoldiella* may encompass species from other genera due to its small size and the potential for confusion with immature specimens. La Perna (2004) observed that, based on the traditional description at the time, *Yoldiella* was regarded as a provisional “pigeon-hole”, where numerous species were temporarily allocated in a kind of waiting list, rather than a natural group. It was proposed that within this genus, some clusters of morphologically similar species could be recognized, with the morphological differences among these clusters suggesting distinct systematic ranks. Benaim and Absalão (2011a) followed this conjecture by analyzing the morphological characteristics of some Atlantic species using empty shell specimens and proposed three distinct clusters, grouping together *Y.nana*, *Y.inconspicua*, and *Y.americana* Allen, H.L. Sanders & F. Hannah, 1995. However, in our molecular phylogenetic analysis of these three species, they did not cluster in a single branch. The feasibility of this hypothesis requires validation through the combination of anatomy, morphometry, molecular data, and other methods. In addition, hinge plate features are considered a significant diagnostic feature in descriptions of *Yoldiella* species, especially the width of the posterior hinge plate (Benaim and Absalão 2011b; Benaim et al. 2011). Further meticulous examination of all taxa is essential before achieving confidence in the accuracy of classification.

Based on anatomical and morphological characteristics, Protobranchia has traditionally been regarded as a monophyletic group (Pelseneer 1889; Xu 1999), but the widespread application of molecular methods in phylogenetic analysis has not provided support for the monophyletism of the Protobranchia (Combosch et al. 2017). Recent studies have supported the monophyly of Protobranchia and five superfamilies (Nuculoidea, Manzanelloidea, Solemyoidea, Sareptoidea, and Nuculanoidea) (Smith et al. 2011; Sharma et al. 2012; Bieler et al. 2014; González et al. 2015; Lemer et al. 2019; Sato et al. 2020). Notably, the monophyly of lower taxa (family and below) is still uncertain, particularly within the superfamily Nuculanoidea. In this study, all currently recognized families were found to be non-monophyletic, which is consistent with previous research findings (Sharma et al. 2013; Sato et al. 2020). In addition, the limited support for the majority of nodes within the Nuculanoidea has resulted in largely ambiguous internal relationships. This phenomenon may be the result of a combination of factors, possibly the lack of genetic information, deficient taxon sampling, or both, or even the possibility that this taxon may have undergone rapid radiation (Lemer et al. 2019).

Phylogenetic analysis of the Yoldiidae revealed differences between the evolutionary relationships inferred from the COI and the 18S rRNA genes. Specifically, the phylogenetic tree based on COI gene shows that *Yoldia* is polyphyletic, in which species were interspersed among other genera rather than forming a distinct clade. In contrast, the phylogenetic tree derived from the 18S rRNA gene shows that *Yoldia* is a monophyletic group within a single clade. Such incongruities may be attributed to variations in the evolutionary rates of the two genes. The genetic markers employed in this study, such as COI and H3, exhibit rapid evolutionary rates, particularly at the third codon position, which are almost certainly saturated when applied across multiple taxonomic families. In addition, Mallatt et al. (2010) explored the metazoan tree using almost complete rRNA genes (18S and 28S), which suggested that non-monophyly of Mollusca in analyses based on 18S rRNA might be explained by these non-homologous forms, and indeed paraphyly/polyphyly of Mollusca has been found in many studies of metazoan phylogeny based on 18S rRNA. In addition, Mallatt et al. (2010) investigated the metazoan phylogenetic tree by analyzing nearly complete 18S rRNA genes. Their findings did not support the monophyly of Mollusca, indicating that the non-monophyly of Mollusca in 18S rRNA-based analyses could be attributed to non-homologous forms. Indeed, the paraphyly/polyphyly of Mollusca has been observed in numerous studies on metazoan phylogeny utilizing 18S rRNA (e.g. Winnepenninckx et al. 1996; Mallatt et al. 2010, 2012). Based on the findings derived from the analysis of 18S rRNA, the potential scenario cannot be ruled out. Consequently, great care needed to be taken in the application of 18S rRNA for phylogenetics to avoid the incorporation of non-homologous variants of 18S rRNA in the assessments.

There may be more problems with traditional identification that relies solely on anatomy and morphology. Challenges persist due to the difficulty in obtaining protobranch samples, resulting in relatively limited molecular data with insufficient resolution. As shown in our phylogenetic analysis, the bootstrap values are low which may be unreliable. Nonetheless, this work represented a minor step forward and demonstrated the complexity of the taxonomic classification system within this taxon and made it clear that a greater coverage of taxa and more informative genetic markers (not limited to a single gene, but based on high-throughput sequencing technologies for massive orthologous genes from transcriptomes or even genomes) will provide great potential for improving the resolution of phylogenetic classification in this group in the future.

## Supplementary Material

XML Treatment for Yoldiella

XML Treatment for Yoldiella haimaensis

## ﻿Additional information

### Author ORCIDs

Yan Tang https://orcid.org/0000-0003-0564-7279

Junlong Zhang https://orcid.org/0000-0003-3831-4673

### Data availability

All of the data that support the findings of this study are available in the main text or Supplementary Information.

## Supplementary materials

Supplementary material 1 Primers used in this study Data type xlsx https://binary.pensoft.net/file/1063702This dataset is made available under the Open Database License (http://opendatacommons.org/licenses/odbl/1.0/). The Open Database License (ODbL) is a license agreement intended to allow users to freely share, modify, and use this Dataset while maintaining this same freedom for others, provided that the original source and author(s) are credited.Qi Gao , Yan Tang , Junlong Zhang

Supplementary material 2 The sequences of COI, 18S and H3 genes used for phylogenetic analyses of the superfamily Nuculanoidea Data type xlsx https://binary.pensoft.net/file/1063703This dataset is made available under the Open Database License (http://opendatacommons.org/licenses/odbl/1.0/). The Open Database License (ODbL) is a license agreement intended to allow users to freely share, modify, and use this Dataset while maintaining this same freedom for others, provided that the original source and author(s) are credited.Qi Gao , Yan Tang , Junlong Zhang

Supplementary material 3 The sequences of COI and 18S genes used for phylogenetic analyses of the family Yoldiidae Data type xlsx https://binary.pensoft.net/file/1063704This dataset is made available under the Open Database License (http://opendatacommons.org/licenses/odbl/1.0/). The Open Database License (ODbL) is a license agreement intended to allow users to freely share, modify, and use this Dataset while maintaining this same freedom for others, provided that the original source and author(s) are credited.Qi Gao , Yan Tang , Junlong Zhang

Supplementary material 4 Phylogenetic tree inferred by Bayesian inference (BI) based on combined gene dataset (COI+18S+H3) Data type pdf https://binary.pensoft.net/file/1063705This dataset is made available under the Open Database License (http://opendatacommons.org/licenses/odbl/1.0/). The Open Database License (ODbL) is a license agreement intended to allow users to freely share, modify, and use this Dataset while maintaining this same freedom for others, provided that the original source and author(s) are credited.Qi Gao , Yan Tang , Junlong Zhang

Supplementary material 5 Phylogenetic tree inferred by Maximum likelihood (ML) based on combined gene dataset (COI+18S +H3) Data type pdf https://binary.pensoft.net/file/1063706This dataset is made available under the Open Database License (http://opendatacommons.org/licenses/odbl/1.0/). The Open Database License (ODbL) is a license agreement intended to allow users to freely share, modify, and use this Dataset while maintaining this same freedom for others, provided that the original source and author(s) are credited.Qi Gao , Yan Tang , Junlong Zhang
