# Supplementary material for: ﻿A new species of the genus Yoldiella (Bivalvia, Protobranchia, Yoldiidae) from Haima Cold Seep, South China Sea, China
Source: Zookeys. 2024 Jun 6;1204:223–40. doi: 10.3897/zookeys.1204.121088 (PMC11176815; doi:10.3897/zookeys.1204.121088)
Supplement: Supplementary material 5 — Phylogenetic tree inferred by Maximum likelihood (ML) based on combined gene dataset (COI+18S +H3) [file zookeys-1204-223_article-121088__-s005.pdf]

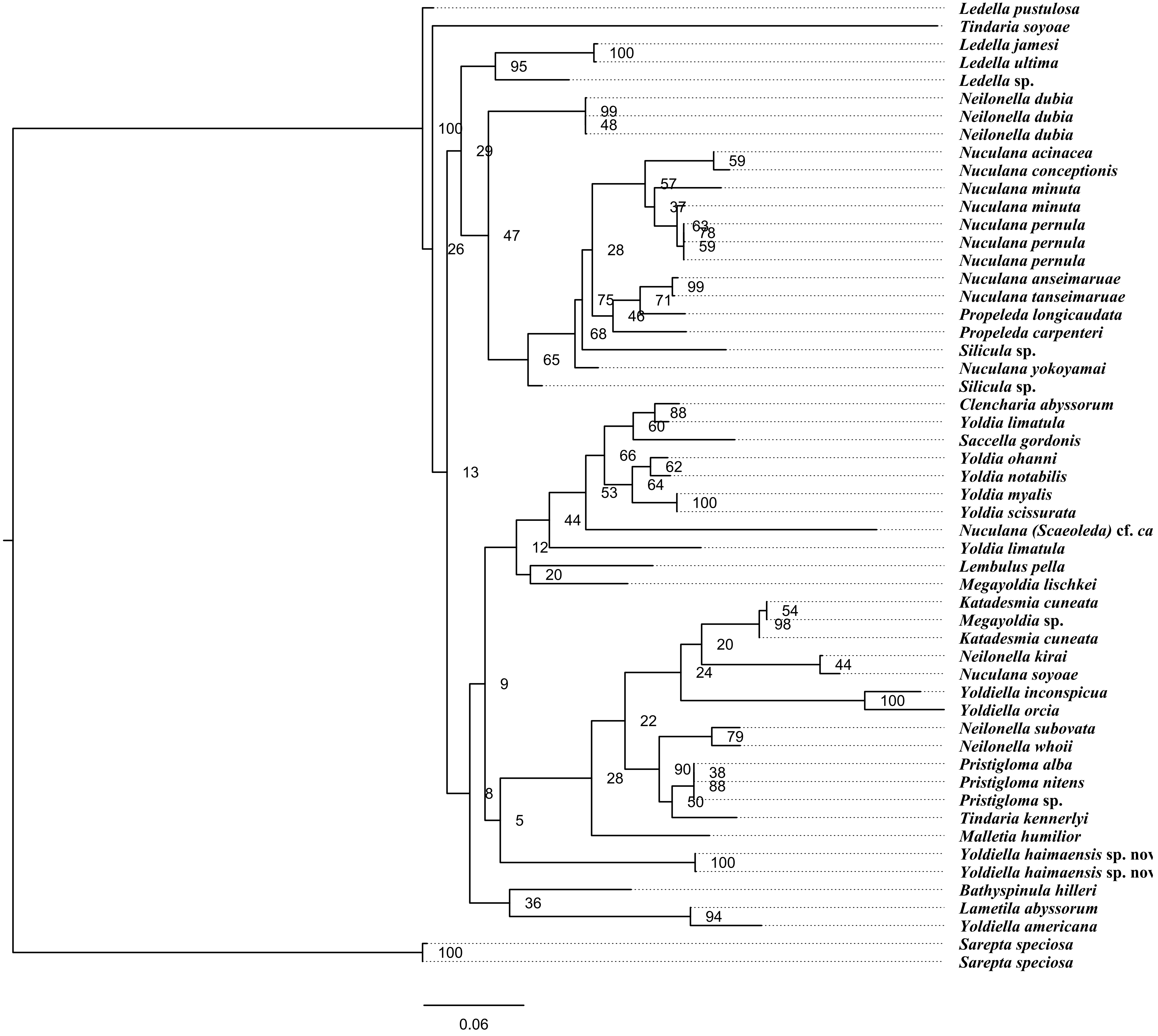

Figure S2: Phylogenetic tree inferred by Maximum likelihood (ML) based on (COI+18S +H3) gene. Numbers adjacent to nodes refer to ML bootstrap scores.
